# An EfficientNet-based hierarchical dual-encoder framework for multi-scale gastrointestinal disease detection

**DOI:** 10.1038/s41598-026-58233-3

**Published:** 2026-06-19

**Authors:** Routhu Srinivasa Rao, Dasari Siva Krishna, Kolipakula Jhatesh Gupta, Bumshik Lee

**Affiliations:** 1 Department of Information and Communication Engineering, Chosun University, Gwangju, 61452 Republic of Korea; 2 Department of Computer Science and Engineering, Gandhi Institute of Technology and Management, Visakhapatnam, Andhra Pradesh 530045 India; 3 JPMorgan Chase, Bengaluru, Karnataka India; 4 Energy AI, Korea Institute of Energy Technology (KENTECH), Naju, Republic of Korea

**Keywords:** Gastrointestinal, Efficientnet, Deep learning, Classification, Computational biology and bioinformatics, Engineering, Gastroenterology, Mathematics and computing

## Abstract

Gastrointestinal (GI) diseases represent a major global health burden, making accurate and early diagnosis critical for improved clinical outcomes. We propose a dual-backbone convolutional framework that integrates EfficientNet-B0 and EfficientNet-B4 to jointly capture fine-grained local details and high-level global context in endoscopic images. The two feature streams are fused through residual learning with channel expansion–reduction and 1*×*1 convolutions, followed by a Convolutional Block Attention Module (CBAM) that adaptively emphasizes diagnostically relevant regions while suppressing background noise. To improve generalization and training stability, MixUp augmentation and Stochastic Weight Averaging (SWA) are employed, and a dropout-regularized classifier is used to mitigate overfitting. Experimental results demonstrate that the proposed method achieves an overall accuracy of 84.11% and a Macro-F1-score of 72.11%, consistently outperforming single-backbone EfficientNet variants and other baseline models, validating the effectiveness of the proposed architecture in both performance and efficiency.

## Introduction

The gastrointestinal (GI) system is a complex network of organs responsible for digestion and nutrient absorption, playing a critical role in maintaining overall health. However, this vital system is vulnerable to various disorders, including polyps, esophageal diseases, colon cancer, and ulcerative colitis, significantly affecting the stomach, intestines, liver, and pancreas. Over the past two decades, advancements in medical imaging have revolutionized the diagnosis of GI diseases, yet accurate and early detection remains a pressing challenge. Early diagnosis is paramount for effective treatment but often requires significant medical expertise, making the process costly, time-intensive, and prone to human error. Furthermore, rural and underprivileged areas frequently lack access to skilled medical professionals, exacerbating challenges in timely disease detection and management^1,2^. The global burden of GI disorders is substantial, with diseases such as colorectal cancer ranking among the most prevalent and deadly conditions worldwide. In the United States alone, approximately 132,000 new cases of colorectal cancer are diagnosed annually. Globally, GI malignancies accounted for 4.8 million new cases and 3.4 million deaths in 2018, including significant contributions from stomach, liver, and esophageal cancers^3,4^. The high prevalence of these conditions underscores the urgent need for advanced diagnostic methods that mitigate mortality rates through early detection and intervention.

Endoscopic procedures, including esophagogastroduodenoscopy (EGD) and colonoscopy, remains the gold standard for diagnosing GI disorders by enabling direct visualization of the GI tract. Additional imaging modalities such as capsule endoscopy, endoscopic ultrasonography (EUS), computed tomography (CT), magnetic resonance imaging (MRI), and positron emission tomography (PET) have further expanded diagnostic capabilities. However, despite these advancements, traditional endoscopy methods face challenges in navigating the intricate structure of the small intestine, often resulting in missed lesions or incomplete diagnoses. Notably, polyp misdetection rates during colonoscopy range from 21.4% to 26.8%, highlighting significant gaps in diagnostic accuracy^5,6^. To address these challenges, wireless capsule endoscopy (WCE) was introduced as a transformative innovation, enabling non-invasive imaging of the GI tract. Patients swallow a capsule equipped with a wireless camera, light source, and radiofrequency emitter, which autonomously captures thousands of high-resolution images as it traverses the digestive tract. These images are compiled into a video for review by medical experts. While WCE offers a more comfortable and detailed diagnostic approach compared to traditional endoscopy, the process is labor-intensive and time-consuming, generating over 50,000 images per procedure that require hours of analysis by clinicians. This increases the risk of diagnostic errors and delays^7,8^.

Recent advancements in artificial intelligence (AI), particularly deep learning-based methods^9,10^, have demonstrated immense potential in automating medical image analysis. Convolutional neural networks (CNNs) have proven highly effective in extracting hierarchical features for classifying and segmenting GI tract abnormalities with greater accuracy^11,12^. Numerous studies have explored hybrid network designs and attention mechanisms to improve endoscopic image analysis. However, challenges such as low-resolution imaging, varying lighting conditions, and the subtle appearance of abnormalities still hinder robust and reliable disease detection^13,14^.

To overcome these limitations, various AI-driven frameworks^15^ and^16^have been introduced. For instance^17,18^, proposed a Local-Global Convolutional Neural Network (LG-CNN) combined with an Endoscopy Lesion Attention (ELA) module and a Gastrointestinal Endoscopy CNN (GE-CNN). The LG-CNN extracts multi-scale features essential for precise classification, while the ELA module enhances focus on relevant regions, reducing the influence of irrelevant details. These innovations significantly improve diagnostic accuracy, minimize analysis time, and provide valuable clinical support. Additionally, transformer-based architectures have been explored for endoscopic image analysis, with studies by Subedi et al. (2024)^15^ demonstrating enhanced feature extraction and classification performance.

Building on these advancements, the proposed work presents a novel architecture, DualEncoderNet, specifically designed for the multi-class classification of gastrointestinal diseases using a comprehensive multi-class endoscopy image dataset. This framework bridges the gap between computational efficiency and high accuracy, providing a promising solution for computer-aided gastrointestinal disease detection.

The following are the significant contributions of this work:

- We propose a novel dual encoder architecture combining EfficientNet-B0 and EfficientNet-B4 to effectively capture both fine-grained local features and high-level global representations enhancing GI disease classification performance.
- To facilitate seamless multi-scale feature fusion, we propose a dual-stage fusion mechanism incorporating a Channel Expansion module and Channel Reduction module. We further integrate a Convolutional Block Attention Module (CBAM) to refine fused features, enhancing the model’s sensitivity to subtle gastrointestinal anomalies.
- We implement a robust training strategy utilizing Stochastic Weight Averaging (SWA) and Test-Time Augmentation (TTA), which significantly boosts model reliability and achieves a real-time inference speed of 153 FPS on GPU.
- By effectively combining local and global feature extraction, our framework demonstrates competitive performance for GI disease detection, significantly improving classification performance in medical imaging applications.

The remainder of this paper is structured as follows: Section 2 presents a review of related literature, Section 3 details the proposed methodology, Section 4 describes the experimental setup and results, and Section 5 concludes the study with future research directions.

## Related work

Recent advancements in artificial intelligence (AI) and deep learning have significantly enhanced the accuracy and efficiency of gastrointestinal (GI) disease diagnosis through endoscopic imaging. Researchers have proposed innovative models, frameworks, and datasets to address challenges such as inter-class similarities, intra-class differences, and the computational complexity of traditional methods.

Montalbo (2022)^11^ introduced the Multi-Fused Residual Convolutional Neural Network (MFuRe-CNN), integrating auxiliary fusing layers and alpha dropouts to reduce overfitting and improve accuracy. This model achieved an impressive 97.25% accuracy while maintaining efficiency with only 4.8 million parameters and 7.8 GFLOPs, showcasing a cost-effective alternative to conventional approaches. Khan et al. (2024)^5^ designed a multi-module attention-guided deep learning framework that combines local-global feature extraction, lesion-specific attention, and a dedicated classification module. Their framework successfully addressed inter-class and intra-class challenges, demonstrating robust performance on the Kvasir and HyperKvasir datasets. Mohapatra et al. (2023)^19^ employed empirical wavelet transform (EWT) to preprocess endoscopic images and decompose them into informative modes, subsequently using deep convolutional neural networks (CNNs) for disease classification. Their two-level classification approach achieved accuracies of 96.65% and 94.25% in separate stages, emphasizing the utility of EWT in enhancing feature representation. Datasets have also been critical to advancing GI disease diagnosis. Jha et al. (2023) developed GastroVision, a large-scale, multi-class endoscopic image dataset annotated by expert endoscopists. This resource facilitates benchmarking for deep learning models and addresses the scarcity of diverse labeled datasets in GI research.

Several works have focused on leveraging attention mechanisms to improve model performance. Ye et al. (2024)^12^ proposed an SE+ResNet-H model that combines Squeeze-and-Excitation blocks with transfer learning to achieve an accuracy of 98.47%. Similarly, Ahmad et al. (2023)^6^ enhanced YOLOv7 for real-time gastric lesion detection using attention modules, achieving high precision and speed suitable for clinical use. Ensemble approaches have also gained traction. Gunasekaran et al. (2023)^18^ introduced GIT-Net, an ensemble model that combines DenseNet201, InceptionV3, and ResNet50 using weighted averaging. Their method outperformed individual models with a classification accuracy of 95% on the KVASIR v2 dataset. Other studies have explored optimization techniques to improve classification. Mirza et al. (2023)^20^ employed Hybrid Rice Optimization for hyperparameter tuning in their hybrid deep learning framework, achieving enhanced accuracy for cancer classification. Khan et al. (2020)^3^ integrated feature fusion with Particle Swarm Optimization (PSO) for stomach infection recognition, demonstrating exceptional results with a private dataset. Liedlgruber and Uhl (2011)^1^ provided a foundational review of computer-aided decision support systems for endoscopy, highlighting the evolution of imaging technologies and challenges in clinical adoption.

Iakovidis et al. (2018)^7^ proposed a novel weakly supervised methodology for automatic detection and localization of GI anomalies in endoscopic video frames. The technique involved a three-phase framework: classifying frames as normal or abnormal using a weakly supervised CNN (WCNN), detecting salient points with a saliency detection algorithm, and localizing anomalies using an iterative cluster unification (ICU) algorithm. This approach demonstrated high efficacy, achieving an area under the receiver operating characteristic (AUC) of over 80%, with the highest anomaly detection AUC reaching 96% for gastroscopy images and 88% for wireless capsule endoscopy images. Habe et al. (2024)^8^ conducted an extensive benchmark of object detection models for WCE pathology detection. They evaluated models such as Single Shot Multibox Detector (SSD), YOLOv3, and semi-supervised versions of Faster R-CNN on a dataset derived from the Kvasir-Capsule repository. The results highlighted the efficiency of RTMDet-S and SSD300 models, achieving over 99% accuracy, while YOLOv3 provided the best operational efficiency at 92.1 frames per second. These findings underline the potential of semi-supervised approaches in enhancing sensitivity and specificity for GI anomaly detection. Noor et al. (2023)^17^ tackled the challenges of low-contrast WCE images by proposing a brightness-controlled contrast-enhancement method optimized with a genetic algorithm. Their method significantly improved image quality and, consequently, the performance of pretrained CNN models used for classification. The proposed framework demonstrated substantial accuracy improvements–15.26% in accuracy, 16.77% in recall, and 15.18% in F-measure–showcasing its superiority over state-of-the-art techniques.

Alruban et al. (2023)^21^ introduced a novel technique, EIAGTD-NIADL, which combines nature-inspired optimization algorithms with deep learning models for GI disease detection. The method used bilateral filtering for preprocessing, an enhanced ShuffleNet for feature extraction, and a spotted hyena optimizer for further optimization. The stacked LSTM classifier further improved the diagnostic accuracy, outperforming existing methods when validated on benchmark datasets. Elmagzoub et al. (2024)^22^ emphasized the integration of attention mechanisms with transfer learning models for GI disease classification. By fine-tuning ResNet101 with grid search optimization and integrating an attention mechanism, they achieved an accuracy of 93.5%. This approach effectively focused on diagnostically relevant image regions, enhancing the overall classification performance. Kundu et al. (2020)^13^ developed a unified scheme for detecting multiple GI diseases using WCE images. Their method employed a least-square saliency transformation (LSST) for identifying salient regions, followed by a probabilistic model-fitting approach for feature extraction. This scheme showed superior performance compared to existing methods, providing a robust solution for multi-disease classification.

Obayya et al. (2023)^23^ presented the Modified Salp Swarm Algorithm with Deep Learning (MSSADL-GITDC) for GI disease classification. By integrating median filtering, a modified Capsule Network with class attention layers, and a deep belief network with extreme learning machines, the model achieved a remarkable accuracy of 98.03% on the Kvasir-V2 dataset. This highlights the potential of combining optimization algorithms with deep learning for medical imaging. Du et al. (2019)^2^ reviewed the applications of deep learning in GI endoscopy, highlighting advancements in disease detection, classification, segmentation, and localization. The study also identified key challenges, such as the need for annotated datasets and the complexity of handling variability in endoscopic images, suggesting directions for future research. Agrusa et al. (2022)^24^ explored robust regression and optimal transport methods to analyze electrogastrograms (HR-EGG) for differentiating GI disease etiologies. Their model effectively predicted symptom severity and offered insights into tailoring treatment strategies, addressing unmet needs in non-invasive diagnostics. Zhang et al. (2022)^25^ introduced a secondary quality evaluation model based on the QoS and QoE framework of 5G networks for improving image recognition accuracy in GI disease diagnosis. This approach significantly enhanced diagnostic precision, achieving an accuracy of 98.5%, showcasing its potential for integrating network advancements with medical imaging.

Wafa et al. (2024)^26^ proposed a deep learning-based framework that employs histopathology images to classify MSI and MSS. Their comprehensive methodology includes data acquisition, image preprocessing, feature extraction, and classification. Notably, a hybrid model combining convolutional neural networks (CNNs) with RNN variants outperformed others, achieving an impressive accuracy of 99.9%, highlighting its potential in early GI cancer detection. Maurício and Domingues (2024)^27^ explored the use of deep learning in differentiating Crohn’s disease from ulcerative colitis, leveraging datasets like LIMUC and HyperKvasir. Their approach incorporated CNNs and Vision Transformers (ViTs) and utilized knowledge distillation to create lightweight models suitable for hospital deployment. They demonstrated that it is possible to reduce model parameters by 25x while maintaining high accuracy. Their interpretability analyses ensured that the distilled models retained the same performance as the original architectures, providing a practical solution for real-time medical diagnostics. Khan et al. (2019)^14^ addressed the challenge of processing large volumes of wireless capsule endoscopy (WCE) data by proposing a saliency-based method in the YIQ color space. Their approach included HSI color transformation, segmentation, and feature fusion using discriminant techniques such as SVD, LBP, and GLCM. With a dataset of 9,000 WCE images, they achieved improved segmentation and classification accuracy, emphasizing the importance of pre-processing and feature extraction in automated diagnostics.

Raju et al. (2024)^28^ introduced GIEnsemformerCADx, a hybrid ensemble approach combining vision transformers, CNNs, and bidirectional LSTMs. This system utilized datasets such as HyperKvasir and CKHK-22 to detect colorectal cancer. Their innovative fusion models, including DaRD-22, achieved superior accuracy rates of 93.3% and 91.67% on these datasets, respectively, demonstrating the potential of hybrid architectures in enhancing diagnostic precision. Waheed and Gui (2024)^29^ implemented an ensemble learning model optimized with a cuckoo search algorithm for GI disease detection. By combining DenseNet169, InceptionV3, and MobileNet, their approach achieved an outstanding accuracy of 99.75% on a dataset of 6,000 endoscopic images. The inclusion of Grad-CAM analysis provided insights into model decision-making, emphasizing transparency and interpretability in diagnostic systems. Jiang et al. (2024)^4^ reviewed the application of DL methods in computer-aided diagnosis (CAD) systems for GI diseases. Their survey addressed challenges in clinical diagnosis, such as complex imaging scenes and the need for multi-class disease classification. They outlined a stepwise process for organ localization, single disease classification, and multi-class disease detection, emphasizing the importance of integrating these steps into an effective CAD system. Bhardwaj et al. (2023)^30^ employed deep transfer learning models to classify GI diseases using endoscopic images from the Kvasir dataset. Their study demonstrated that InceptionResNetV2 and Xception models provided the highest accuracy for specific disease classes, achieving results as high as 98.88%. The findings underscore the significance of selecting appropriate pre-trained architectures for specific disease classifications.

Demirbaş et al. (2024)^31^ proposed the Spatial Attention ConvMixer (SAC) architecture, which enhances ConvMixer with spatial attention mechanisms. This system assigns importance to spatial locations in feature maps, improving classification accuracy on the Kvasir dataset to 93.37%. Their comparison with models like Vanilla ViT and ResNet50 confirmed the efficacy of spatial attention in automated GI disease detection. Han et al. (2024) introduced a curriculum self-supervised learning framework for GI endoscopy image classification. Utilizing the HyperKvasir dataset, their approach achieved a top-1 accuracy of 88.92%, demonstrating the potential of self-supervised learning in leveraging large unlabeled datasets to improve diagnostic outcomes. Alshardan et al. (2024)^32^ developed the Harbor Seal Whiskers Optimization Algorithm (HSWOA) integrated with deep learning for GI cancer detection. Using Xception for feature extraction and XGBoost for classification, their method achieved remarkable accuracy. The inclusion of moth-flame optimization for parameter selection further enhanced the system’s performance.

Zeng et al. (2024)^33^ developed a deep learning-based survival prediction model for Gastrointestinal Stromal Tumor (GIST) patients using the SEER database. Their DeepSurv model outperformed traditional methods, demonstrating higher accuracy and better risk stratification compared to the AJCC staging system, as validated by metrics like the C-index, AUC, and NRI. Wang et al. (2024)^34^ proposed MCH-PAN, a model for detecting gastrointestinal polyps. By integrating multi-scale features and a channel attention mechanism, they addressed the challenge of polyp scale variation, achieving improved detection accuracy and reduced diagnostic uncertainty. Barki et al. (2024)^35^ explored superpixel-based segmentation methods, using SLIC and LSC to enhance feature extraction from GI images. Their results showed that superpixel-based techniques outperformed traditional methods in both accuracy and efficiency, particularly for middle and lower GI tract sections. Wang et al. (2023)^36^ introduced a Vision Transformer model with hybrid shifted windows for gastrointestinal endoscopy image classification. This model successfully captured both short- and long-range dependencies in complex GI images, achieving high classification accuracy on the Kvasir v2 and HyperKvasir datasets. Recent advancements have also explored the use of large-scale vision foundation models for gastrointestinal image analysis. Mao et al. (2025)^37^ proposed a one-shot polyp segmentation framework based on the Segment Anything Model (SAM), incorporating cascaded prior generation and iterative prompt evolution to enable accurate segmentation under extremely limited annotation settings. This approach demonstrates the potential of foundation models to generalize across diverse polyp appearances and highlights a shift toward prompt-based and data-efficient learning paradigms in medical imaging. However, such approaches are primarily focused on segmentation and prompt-driven inference, whereas our proposed DualEncoderNet is designed for robust multi-class classification by leveraging heterogeneous backbone fusion and attention-guided feature refinement.

Beyond imaging-based analysis, recent computational studies have explored disease mechanisms using genetic optimization and interactome-based modeling approaches. For example, Zhu et al. (2024)^38^ proposed an ant colony optimization-guided genetic framework to identify gene associations in Alzheimer’s disease, highlighting the utility of computational intelligence in uncovering complex gene-disease relationships. Similarly, Sahrawat et al. (2025^39^) employed an interactome-based network approach to analyze associations between cardiomyopathy and cardiovascular diseases, demonstrating how protein–protein interaction modeling can reveal shared pathogenic pathways. These systems-level methodologies complement imaging-based phenotypic analysis and underscore the importance of integrating multimodal computational frameworks for comprehensive disease understanding.

## Proposed method

**Fig. 1:**
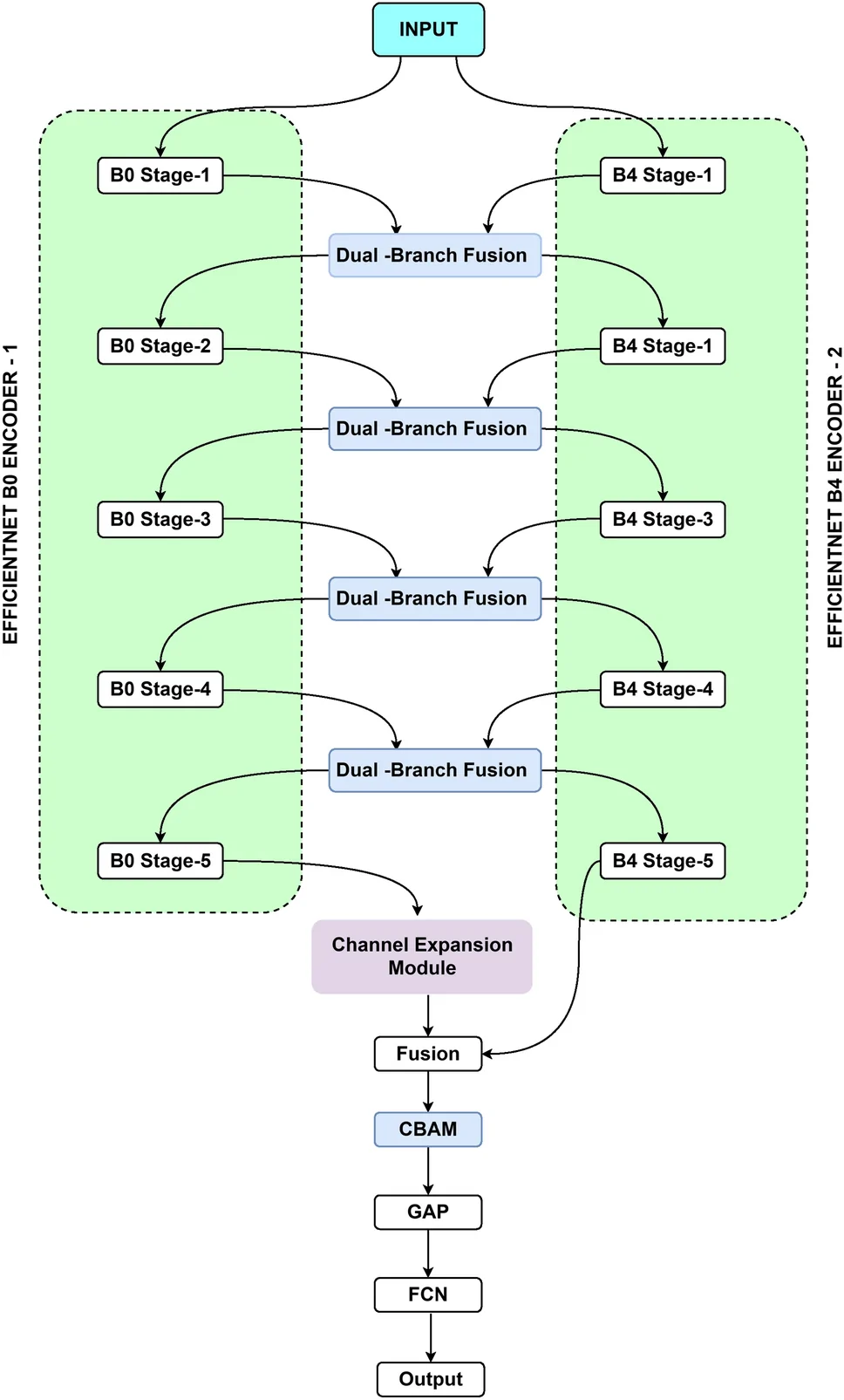
The architecture of the proposed model.

In this work, we introduce a novel dual-encoder framework combining two variants of EfficientNet–EfficientNet-B0 and EfficientNet-B4–as parallel feature extraction modules.The design leverages the complementary strengths of these encoders to extract robust and multiscale features from input images. The input to the system is a preprocessed image, resized to match the input dimensions required by the EfficientNet models. Standard data augmentation techniques, such as normalization and random flipping, are applied to enhance the model’s generalization capability. The architecture of the proposed model is shown in Figure 1 and working of the model is shown in Algorithm 1.

**Algorithm 1:**
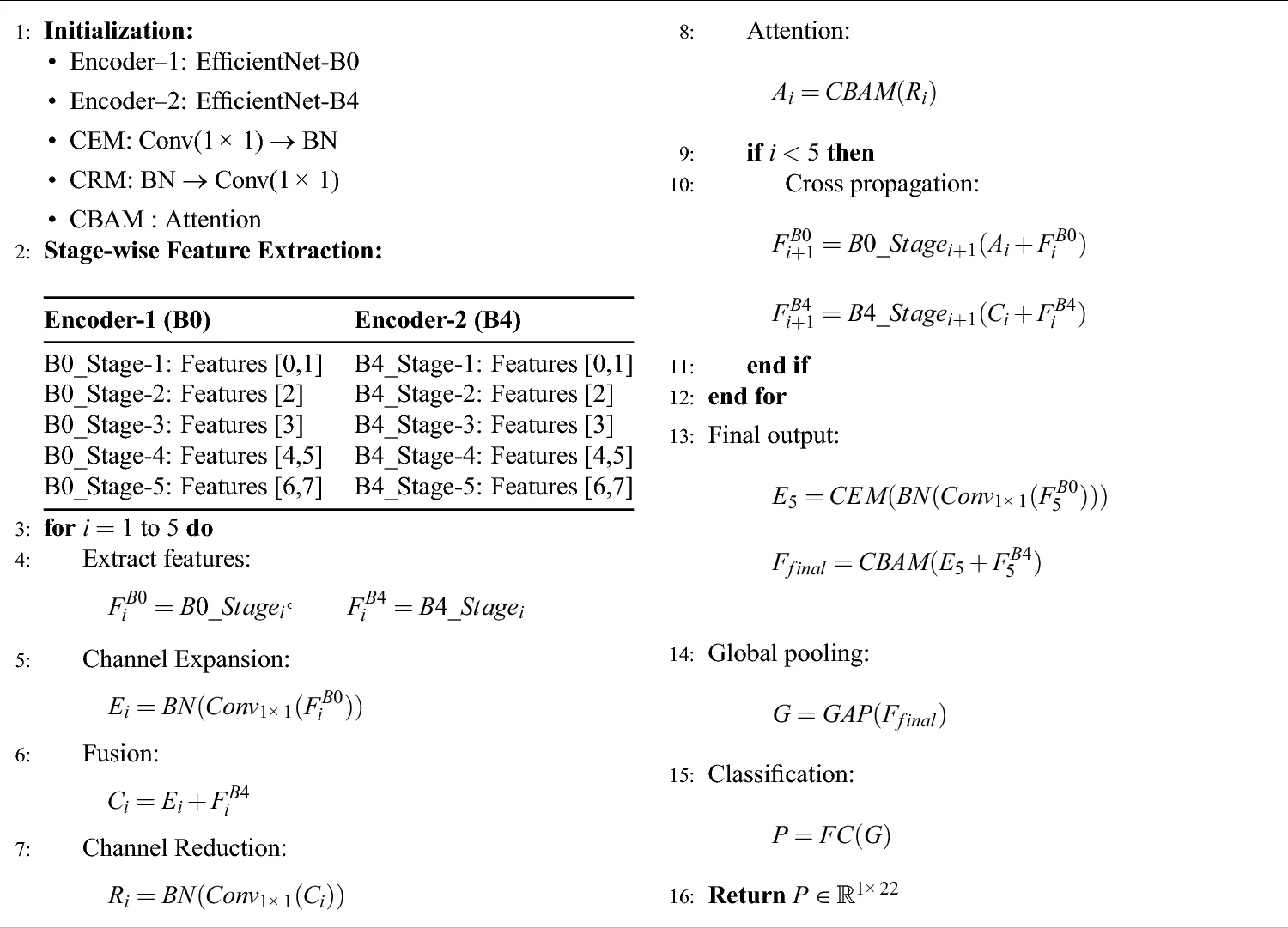
Dual encoder architecture with dual-branch fusion, CEM, CRM, and CBAM

### Dual encoder architecture

The proposed architecture comprises two parallel encoders, EfficientNet-B0 and EfficientNet-B4, as shown in Figure 1. The EfficientNet-B0 encoder, a lightweight variant, extracts features at a lower computational cost by passing the input through a series of convolutional blocks (B0 Stage-1 to B0 Stage-5). Simultaneously, EfficientNet-B4 processes the input with a deeper network structure, capturing richer semantic information.

At each stage, the feature maps from EfficientNet-B0 and EfficientNet-B4 are fused using a dual-stage fusion mechanism. This fusion effectively integrates fine-grained details from B0 with high-level semantic representations from B4, enhancing the overall feature representation for improved performance. Combining these two encoders ensures an optimal balance between computational efficiency and representational power. To address potential concerns regarding spatial misalignment, we clarify that the fusion is performed only between stage pairs that naturally share the same spatial resolution. EfficientNet-B0 and EfficientNet-B4 follow identical downsampling schedules at corresponding stages, ensuring that $B0_{Si}$ and $B4_{Si}$ always have matching height and width. Therefore, no interpolation or pooling is required for spatial alignment; only channel alignment is performed using a *1 × 1* convolution before fusion.

Furthermore, the Convolutional Block Attention Module (CBAM)^40^ attention mechanism and skip connections facilitate feature reuse and enable the network to capture hierarchical representations, preserving important spatial information across different layers. The multi-scale feature extraction capability, enabled by the two encoders operating at different capacities, allows the model to capture both global and local features effectively, improving its ability to generalize across various gastrointestinal disease detection.

The selection of EfficientNet-B0 and EfficientNet-B4 was motivated by their heterogeneous representational capacities. EfficientNet-B0, being lightweight, is effective at capturing fine-grained local texture patterns, while EfficientNet-B4, with deeper layers and higher channel capacity, encodes richer semantic abstractions. This heterogeneous pairing encourages complementary feature learning rather than redundancy. Empirical ablation results demonstrate that pairings with closer-capacity backbones (e.g., B1+B4 or B2+B4) provide diminished returns in macro-level performance and correlation metrics, suggesting that representational diversity between encoders plays a critical role in improving class-level discrimination.

**Fig. 2:**
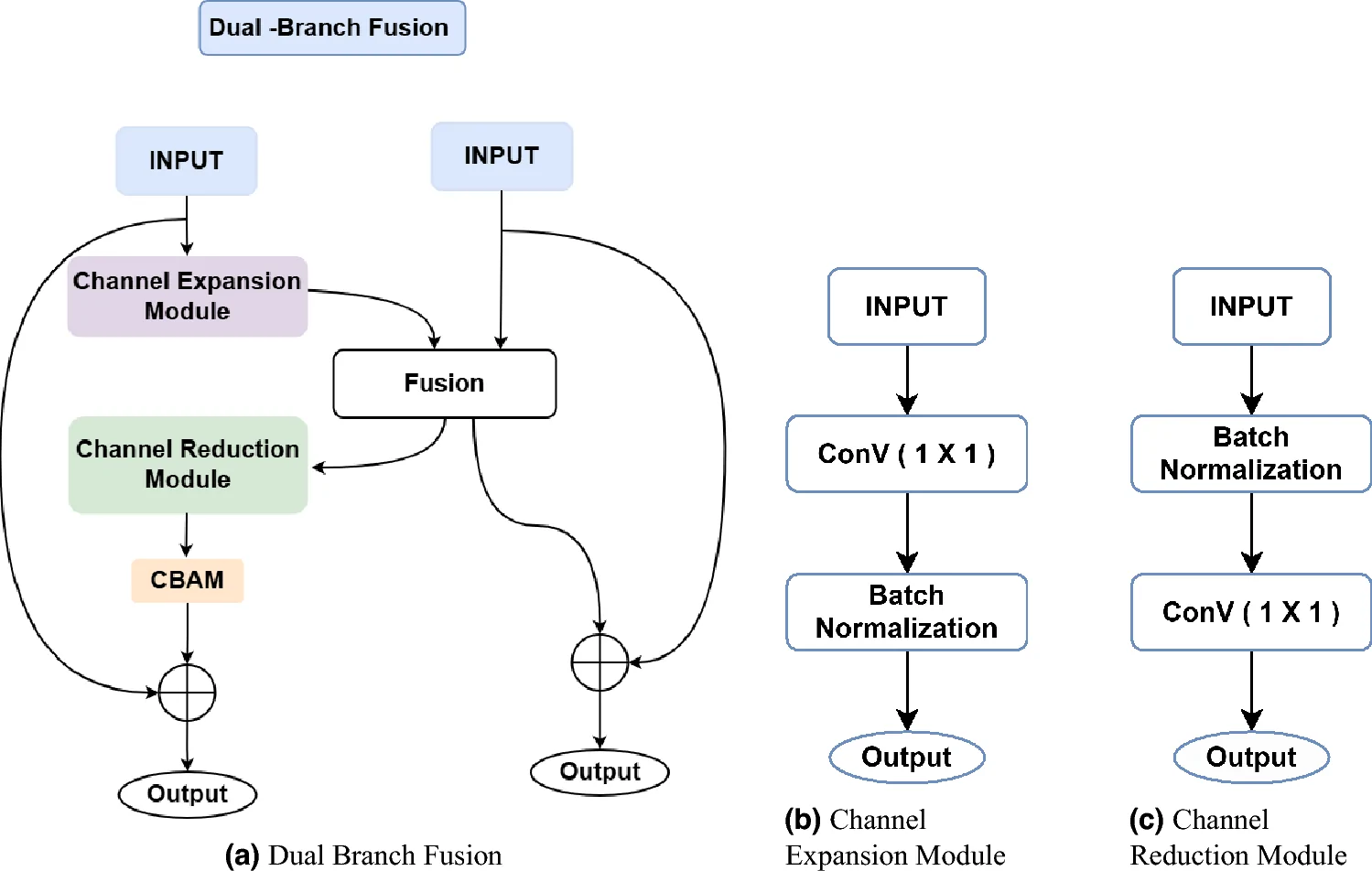
Overview of the proposed DualEncoderNet fusion mechanism.

### Dual branch fusion

The Dual-Branch Fusion mechanism, illustrated in Fig. 2(a), integrates feature representations from parallel encoders using two key components–Channel Expansion Module (CEM) and Channel Reduction Module (CRM)–forming a structured hierarchical fusion pipeline rather than simple feature aggregation. Unlike conventional late-fusion strategies that combine representations only at the final stage, the proposed mechanism enables progressive inter-branch interaction across multiple hierarchical levels, allowing fine-grained textures and high-level semantic representations to be aligned throughout the network.

At each stage, feature maps from EfficientNet-B0 are first passed through the Channel Expansion Module (CEM), which projects them into a higher-dimensional space to match the channel dimensionality of EfficientNet-B4. This projection ensures compatibility for fusion while enhancing representational richness. Additionally, it mitigates the risk of semantic dominance from the higher-capacity backbone during feature aggregation, promoting balanced contribution from both encoders. The expanded features are then combined with corresponding features from the parallel encoder in the Fusion module, and the fused output subsequently passes through the Channel Reduction Module (CRM) to compress the channels back to EfficientNet-B0’s original dimensionality so that the next stages can process them without mismatch. This expansion–reduction cycle stabilizes feature interaction across heterogeneous backbones while maintaining computational efficiency.

To further refine the fused representations, a Convolutional Block Attention Module (CBAM)^40^ is applied after the reduction step. CBAM sequentially performs channel and spatial attention to emphasize diagnostically relevant regions while suppressing irrelevant responses. The refined features are then propagated to subsequent stages through residual connections, enabling effective reuse of both local and global contextual information and improving the model’s discriminative capability.

#### Channel expansion module

The Channel Expansion Module (CEM), detailed in Fig. 2(b), is designed to enhance the feature representation by increasing the number of channels in a feature map. This process expands the feature channels of EfficientNet-B0 to match those of EfficientNet-B4, ensuring compatibility before fusion. By applying a 1*×*1 convolution followed by batch normalization, this module performs efficient dimensional projection. Beyond dimensional matching, CEM performs semantic calibration by projecting lower-capacity backbone features into the representational space of the deeper encoder, thereby harmonizing feature distributions prior to summation.

In the context of the fusion process, the Channel Expansion Module prepares the input features for integration by aligning and expanding their dimensions to complement other feature maps. This expansion ensures that high-level semantic information is preserved and enriched, which is particularly important in multi-scale or hierarchical feature integration strategies. Furthermore, it ensures compatibility between the two encoders by adapting their feature representations before fusion. This attention mechanism is especially useful for multi-scale feature learning, where capturing both local and global information is critical.

#### Channel reduction module

The Channel Reduction Module (CRM), shown in Fig. 2(c), is designed to reduce the number of channels in a feature map, effectively compressing the feature representation while retaining the most salient information. This step compresses the fused feature maps to match the original EfficientNet-B0 dimensions, ensuring compatibility with its next processing stage. The reduction is achieved using batch normalization followed by a *1 × 1* convolution, which normalizes feature distributions prior to dimensional compression, ensuring stable feature scaling and efficient channel alignment.

In the fusion process, the Channel Reduction Module refines and aligns the fused feature representations, ensuring they are compatible with EfficientNet-B0’s next stage. This reduction step ensures that the model remains lightweight while still preserving key information, preventing redundancy and unnecessary computational overhead. Additionally, CRM prevents channel inflation across successive stages, preserving architectural stability while enabling progressive hierarchical fusion. More importantly, it balances feature contributions when integrating multiple stages or scales, preventing high-dimensional features from dominating the fusion process. This mechanism is particularly effective for multi-stage feature fusion, where concise yet meaningful representations are crucial for robust performance.

**Fig. 3:**
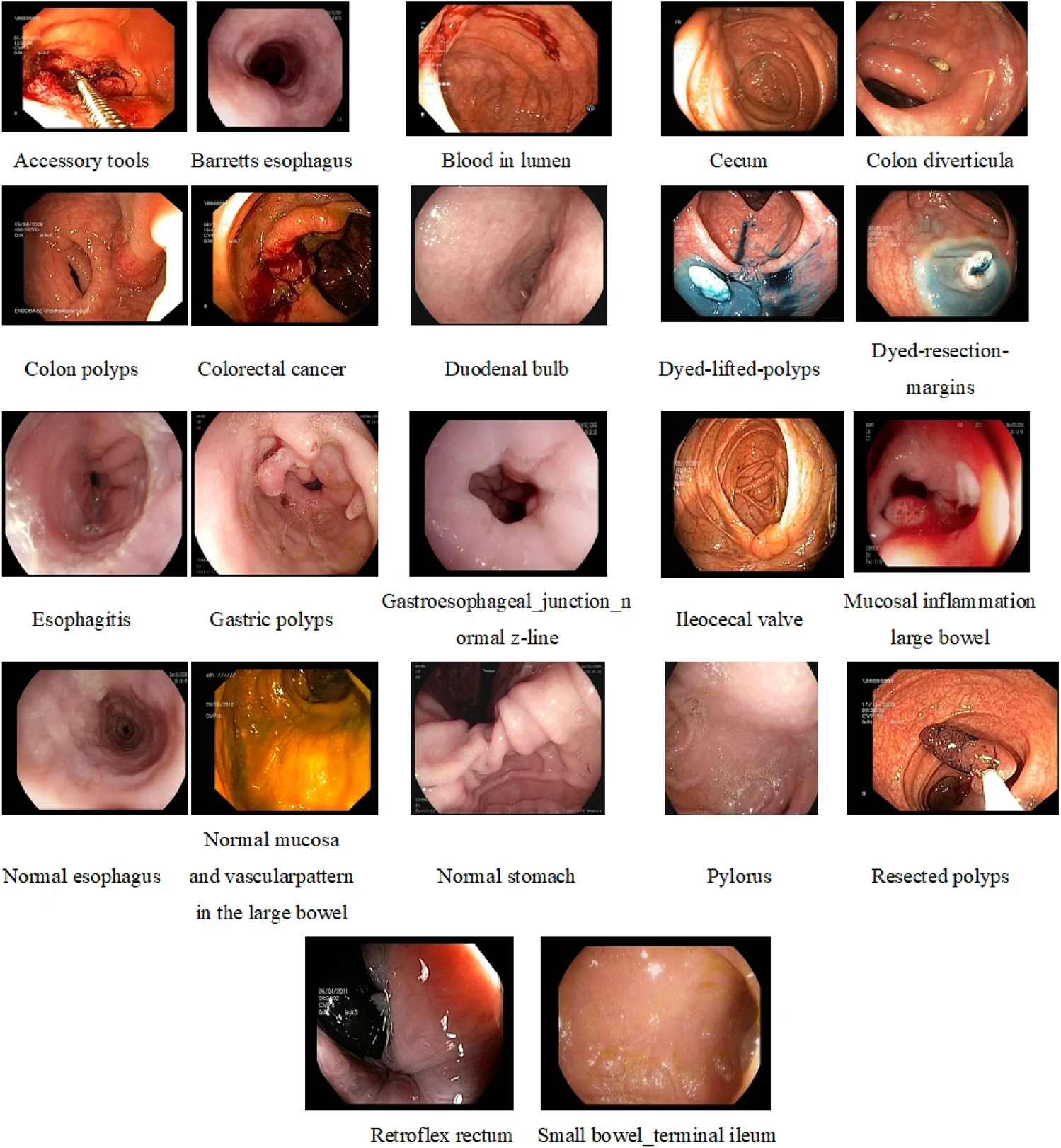
Samples of multi-class endoscopy images.

### Classification

The final classification module processes the fused feature representations obtained from the last stages of EfficientNet-B0 and EfficientNet-B4, ensuring a robust decision-making process. Before final summation, a Channel Expansion Module is applied at the last stage to ensure dimensional compatibility between heterogeneous backbone outputs. This projection aligns channel dimensionalities and stabilizes feature distributions prior to global pooling, preventing representational imbalance at the decision level.

Initially, the fused feature maps undergo a Global Average Pooling (GAP) operation to reduce spatial dimensions while preserving essential information. The pooled features are then passed through a fully connected layer with 256 neurons and ReLU activation, followed by a Dropout layer with a probability of 0.5 to prevent overfitting. Finally, a fully connected output layer with a softmax activation function generates class probabilities for the 22 target classes. This classification strategy ensures that the model effectively utilizes the refined multi-scale features for accurate predictions while maintaining computational efficiency.

### Training strategy and regularization

To mitigate overfitting and enhance the generalization capability of DualEncoderNet, we employed several advanced training strategies. First, we utilized MixUp augmentation with *α =0.1*, which generates synthetic training examples by linearly interpolating between pairs of images and their labels. This encourages the model to behave linearly in-between training examples. Second, we implemented Stochastic Weight Averaging (SWA), which averages the weights of the model traversed by SGD at the end of training (starting from epoch 45). This leads to wider optima and better generalization than standard SGD. Finally, for evaluation, we utilized Test-Time Augmentation (TTA) with four views (original, horizontal flip, vertical flip, and rotation) to average predictions and reduce variance.

## Experimental results

We utilized PyTorch and one GeForce RTX 4090 GPU with 24GB of memory, to train the proposed model efficiently. The dataset, sourced from GastroVision^41^, is a multi-class endoscopy image dataset specifically curated for computer-aided gastrointestinal (GI) disease detection. It offers a comprehensive and diverse collection of endoscopic images, making it an invaluable resource for developing and evaluating deep learning models for automated GI disease classification. The dataset comprises 22 distinct classes, covering a wide spectrum of gastrointestinal conditions.

**Fig. 4:**
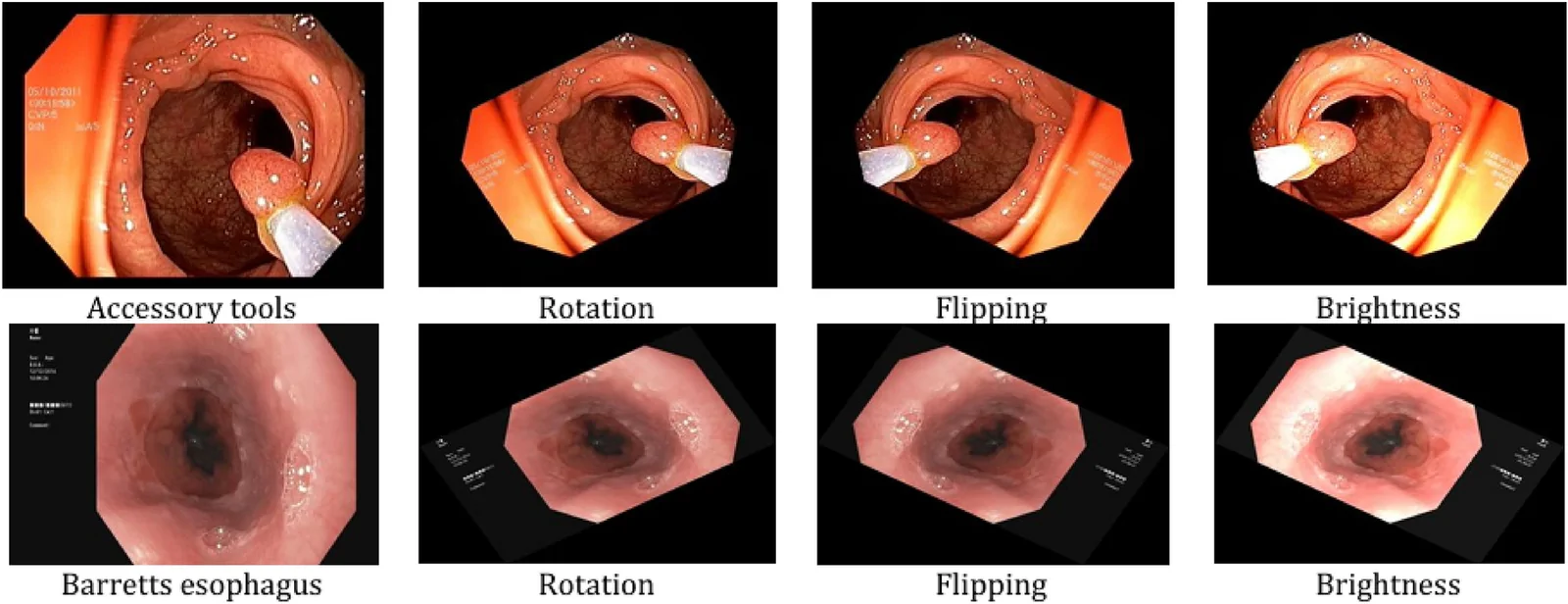
Sample augmentation.

The dataset consists of a total of 7,930 images, distributed as follows: 4,757 images for training, 1,587 for validation, and 1,586 for testing. A detailed class-wise distribution is provided in Table 1, while representative examples of the multi-class endoscopy images are shown in Figure 3. To ensure consistency across the input data, all images were resized to a fixed resolution of 320x320 pixels. Pixel intensity values were normalized to facilitate better convergence during training. Furthermore, data augmentation techniques, including rotation, flipping, zooming, and brightness adjustments, were applied to enhance model generalization. The sample augmentation of rotation, flipping and brightness as shown in Figure 4. The dataset is publicly accessible at https://osf.io/84e7f/.

**Table 1:** Class-wise data distribution.

**Classes**	**Training**	**Validation**	**Testing**	**Total**
Accessory tools	759	254	253	1266
Barretts esophagus	57	19	19	95
Blood in lumen	103	34	34	171
Cecum	68	22	23	113
Colon diverticula	17	6	6	29
Colon polyps	492	165	163	820
Colorectal cancer	84	27	28	139
Duodenal bulb	123	41	41	205
Dyed-lifted-polyps	85	28	28	141
Dyed-resection-margins	147	50	49	246
Esophagitis	64	21	22	107
Gastric polyps	39	13	13	65
Gastroesophageal junction (normal z-line)	198	66	66	330
Ileocecal valve	120	40	40	200
Mucosal inflammation (large bowel)	17	6	6	29
Normal esophagus	84	28	28	140
Normal mucosa and vascular pattern (large bowel)	880	294	293	1467
Normal stomach	581	194	194	969
Pylorus	236	79	78	393
Resected polyps	55	18	19	92
Retroflex rectum	40	13	14	67
Small bowel (terminal ileum)	508	169	169	846

### Performance metrics

To comprehensively evaluate the classification performance, the following metrics were utilized:

1. Micro Precision, Recall, and F1-Score: Aggregates contributions from all classes, treating each instance equally, making it ideal for imbalanced datasets.
2. Macro Precision, Recall, and F1-Score: Calculates metrics for each class individually and then averages them, treating all classes equally.
3. Matthews Correlation Coefficient (MCC): A balanced measure considering true/false positives/negatives, suitable for both binary and multi-class tasks.

The formal definitions of these metrics are provided in Table 2. In this study, we conducted a series of experiments to evaluate the effectiveness of various pretrained models and the proposed hybrid architecture for multi-class gastrointestinal disease detection.

**Table 2:** Evaluation metrics used for multi-class classification.

**Metric**		**Equation**	**Description**
Micro	Precision	$\displaystyle \frac{\text {Total TP}}{\text {Total TP}+\text {Total FP}}$	Micro-averaged metrics compute values globally by aggregating contributions of all classes. They treat each instance equally, making them suitable for imbalanced datasets.
	Recall	$\displaystyle \frac{\text {Total TP}}{\text {Total TP}+\text {Total FN}}$	
	F1-Score	$\displaystyle 2 \times \frac{\text {Precision}_{\text {micro}}\cdot \text {Recall}_{\text {micro}}}{\text {Precision}_{\text {micro}}+\text {Recall}_{\text {micro}}}$	
Macro	Precision	$\displaystyle \frac{1}{n}\sum _{i=1}^{n}\text {Precision}_i$	Macro-averaged metrics compute values for each class independently and then average them. They treat all classes equally, regardless of size. Here, *n* is the number of classes.
	Recall	$\displaystyle \frac{1}{n}\sum _{i=1}^{n}\text {Recall}_i$	
	F1-Score	$\displaystyle \frac{1}{n}\sum _{i=1}^{n}\text {F1-Score}_i$	
Matthews Correlation Coefficient (MCC)		$\displaystyle \frac{\sum _{k}\sum _{l} (C_{kk}C_{ll}-C_{kl}C_{lk})}{\sqrt{\big (\sum _k T_k\big )\big (\sum _k P_k\big )}}$	A balanced metric for binary and multi-class classification. It considers TP, FP, TN, FN and is robust for imbalanced datasets. Here, *C* is the confusion matrix,$T_k$is row sum *k*, and$P_k$is column sum *k*.

### Training configuration

The model was trained with the following specific hyperparameters, chosen to optimize convergence for the dual-backbone architecture:

- **Optimizer:** AdamW (Weight Decay: 0.01)
- **Learning Rate:** Initial LR of 0.0005 with Cosine Annealing Warm Restarts (T_0=10).
- **SWA Schedule:** Stochastic Weight Averaging starts at epoch 45 with a fixed LR of 0.00005.
- **Batch Size:** 16
- **Number of Epochs:** 60
- **Loss Function:** Cross-Entropy Loss with Class Weights and Label Smoothing (0.1)
- **Augmentation:** MixUp (*α =0.1*) and geometric transformations (rotation, flip, affine).

These settings incorporate advanced regularization techniques (MixUp, SWA, Label Smoothing) to prevent overfitting on the complex gastrointestinal dataset.

### Preliminary experiments with pretrained models

The Table 3 presents the performance of various pre-trained models on the Gastrovision dataset. Among the models, ResNet-50 demonstrates the best micro performance with a Precision, Recall, and F1-Score of 0.8146, along with an MCC of 0.7921, indicating strong overall accuracy. DenseNet-169 also performs well, particularly with a micro F1-Score of 0.7055, and maintains decent macro metrics. Swin-T achieves the highest MCC of 0.8191, while models like EfficientNet-B0 and ResNet-152 show lower macro scores. DenseNet-121 balances micro and macro metrics effectively, with an MCC of 0.7987. Other models, including MobileNetV2, Xception, and Inception V3, also demonstrate competitive MCC values, with Swin-T being the standout in terms of overall performance.

We chose to use the EfficientNet model to design our dualencodernet due to its efficient use of computational resources while maintaining competitive performance across various metrics. Despite not achieving the highest MCC or macro F1-Score, EfficientNet-B0 offers a good balance between accuracy and efficiency, making it suitable for scenarios where model size and inference time are critical. Its performance, with a micro F1-Score of 0.6759 and an MCC of 0.6351, ensures that it remains a strong candidate for tasks requiring rapid and resource-efficient processing, without compromising too much on performance. This tradeoff between efficiency and accuracy aligns well with our goals.

**Table 3:** Performance of pre-trained models on the gastrovision dataset.

**Models**	**Micro (%)**			**Macro (%)**			**Accuracy (%)**	**MCC (%)**
	**Precision**	**Recall**	**F1-Score**	**Precision**	**Recall**	**F1-Score**		
ResNet-152^41^	68.79	68.79	68.79	52.58	42.87	44.96	68.79	64.78
EfficientNet-B0^41^	67.59	67.59	67.59	52.85	43.26	45.19	67.59	63.51
DenseNet-169^41^	70.55	70.55	70.55	60.75	46.03	48.83	70.55	66.85
ResNet-50^41^	81.46	81.46	81.46	63.98	60.73	61.76	81.46	79.21
MobileNetV2^15^	73.08	74.00	73.18	-	-	-	74.00	70.83
Xception^15^	74.10	74.99	74.30	-	-	-	74.99	71.95
Inception V3^15^	77.56	78.47	77.74	-	-	-	78.47	76.00
Dendenet201^15^	80.56	81.12	80.46	-	-	-	81.12	78.86
Swin-T^15^	80.75	83.86	83.24	-	-	-	83.86	81.91
DenseNet-121^41^	82.03	82.03	82.03	73.88	62.31	65.04	82.03	79.87

### EfficientNet variants on gastrovision dataset

This section evaluates EfficientNet variants to identify the best backbones for the dual encoder setup. The objective is to determine the most effective combination of EfficientNet models that optimize classification performance while maintaining computational efficiency. The results from the Table 4 show that EfficientNet-B0 performs the best in terms of micro metrics, with a Precision, Recall, and F1-Score of 0.8354, as well as an MCC of 0.8156. However, when comparing the macro metrics, EfficientNet-B0 achieves a Precision of 0.7345, Recall of 0.6520, and F1-Score of 0.6734. EfficientNet-B1 and EfficientNet-B2 show slightly lower performance in both micro and macro metrics, with EfficientNet-B3 demonstrating a decrease in macro performance, particularly in Precision and Recall. Among the larger models, EfficientNet-B6 and EfficientNet-B7 exhibit similar results, with EfficientNet-B7 achieving a macro F1-Score of 0.6716 and an MCC of 0.8045. Based on these results, EfficientNet-B0 and EfficientNet-B4 were identified as the best performing models. EfficientNet-B0 excelled in micro metrics, while EfficientNet-B4 demonstrated strong results across both micro and macro metrics, making them the ideal candidates for setting up the dual encoder in the proposed architecture.

**Table 4:** Pre-trained results of EfficientNet on gastrovision dataset.

**Models**	**Micro (%)**			**Macro (%)**			**Accuracy (%)**	**MCC (%)**
	**Precision**	**Recall**	**F1-Score**	**Precision**	**Recall**	**F1-Score**		
EfficientNet-B0	83.54	83.54	83.54	73.45	65.20	67.34	83.54	81.56
EfficientNet-B1	81.97	81.97	81.97	70.80	65.85	67.43	81.97	79.81
EfficientNet-B2	82.53	82.53	82.53	71.09	63.29	64.86	82.53	80.43
EfficientNet-B3	81.65	81.65	81.65	68.33	64.83	65.65	81.65	79.48
EfficientNet-B4	83.29	83.29	83.29	67.49	63.47	64.88	83.29	81.28
EfficientNet-B5	82.16	82.16	82.16	68.28	62.38	63.68	82.16	80.02
EfficientNet-B6	82.60	82.60	82.60	71.75	65.16	66.83	82.60	80.52
EfficientNet-B7	82.53	82.53	82.53	71.09	65.94	67.16	82.53	80.45

**Table 5:** Performance of dual encoder on gastrovision dataset. Overall accuracy = 84.11 %.

**Classes**	**Precision (%)**	**Recall (%)**	**F1-Score (%)**	**Support**
Accessory tools	94.44	94.07	94.26	253
Barretts esophagus	55.00	57.89	56.41	19
Blood in lumen	83.78	91.18	87.32	34
Cecum	31.25	43.48	36.36	23
Colon diverticula	50.00	66.67	57.14	6
Colon polyps	86.14	87.73	86.93	163
Colorectal cancer	72.00	64.29	67.92	28
Duodenal bulb	75.56	82.93	79.07	41
Dyed-lifted-polyps	77.42	85.71	81.36	28
Dyed-resection-margins	91.49	87.76	89.58	49
Esophagitis	50.00	31.82	38.89	22
Gastric polyps	88.89	61.54	72.73	13
Gastroesophageal junction normal z-line	72.37	83.33	77.46	66
Ileocecal valve	77.27	85.00	80.95	40
Mucosal inflammation (large bowel)	50.00	33.33	40.00	6
Normal esophagus	72.73	85.71	78.69	28
Normal mucosa & vascular pattern (large bowel)	87.27	81.91	84.51	293
Normal stomach	96.53	86.08	91.01	194
Pylorus	78.72	94.87	86.05	78
Resected polyps	35.71	26.32	30.30	19
Retroflex rectum	72.22	92.86	81.25	14
Small bowel terminal ileum	88.17	88.17	88.17	169
**Macro Avg**	72.14	73.30	72.11	1586
**Weighted Avg**	84.55	84.11	84.10	1586

**Table 6:** Performance metrics of the proposed model with unseen data.

Metrics	Proposed model
Micro Precision	84.11%
Micro Recall	84.11%
Micro F1-Score	84.11%
Macro Precision	72.14%
Macro Recall	73.30%
Macro F1-Score	72.11%
MCC	81.91%
Accuracy	84.11%

### Performance of proposed model on gastrovision dataset

The results in the Table 6 demonstrate the performance of the proposed multi-scale EfficientNet-based framework in classifying gastrointestinal (GI) diseases. The framework achieves an overall accuracy of 84.11%, which is a significant achievement given the complexity of the dataset, the variability in GI tract images, and the overlapping features between classes. The Matthews Correlation Coefficient (MCC) of 0.8191 further confirms the balanced performance, showcasing its ability to handle both positive and negative classes well. While the macro F1-Score is 0.7211, which reflects average performance across all classes, the micro metrics are much stronger, emphasizing the model’s ability to achieve consistent results across the most frequent classes.

**Fig. 5:**
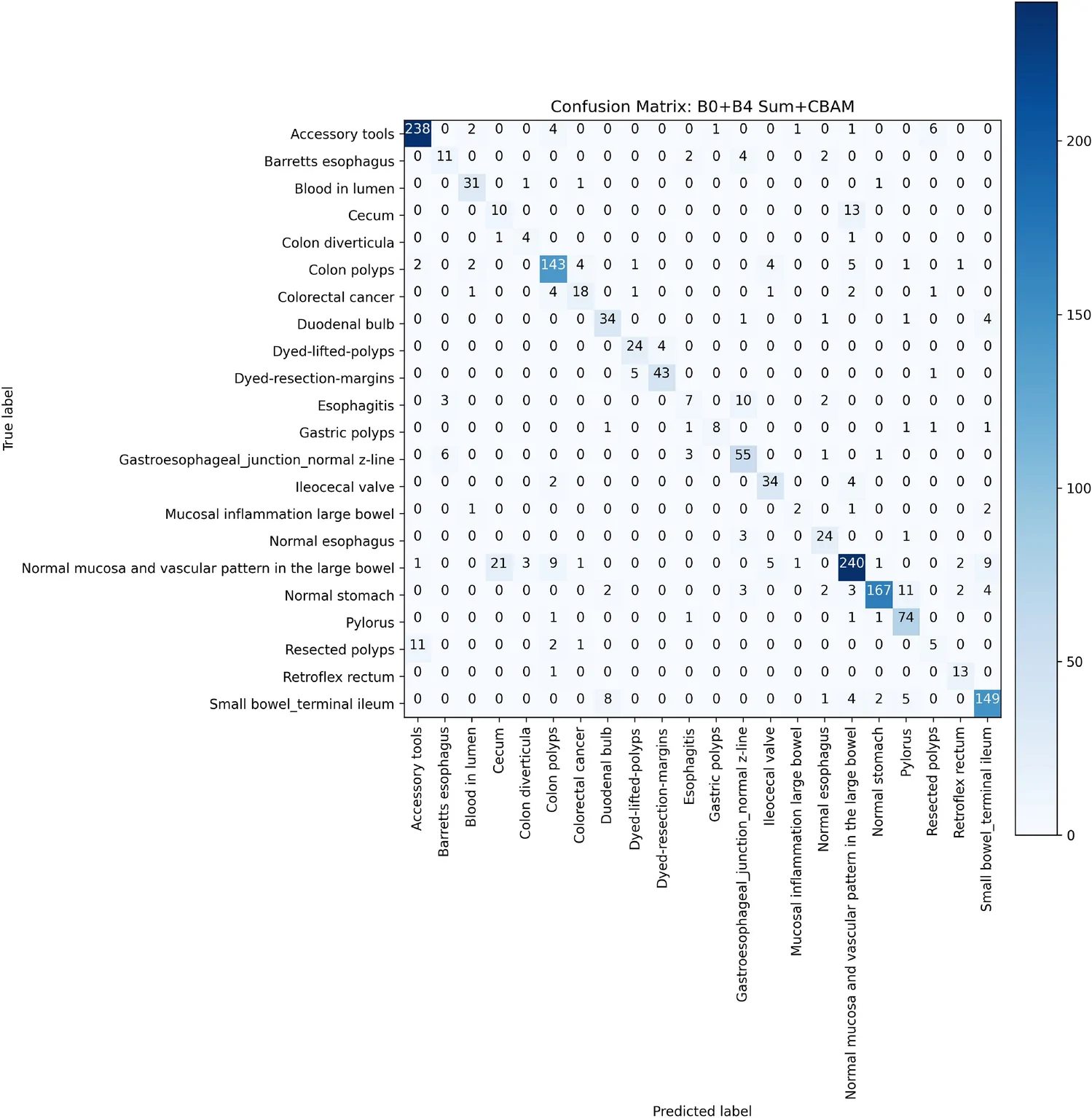
Confusion Matrix of 22 classes on GastroVision dataset.

**Fig. 6:**
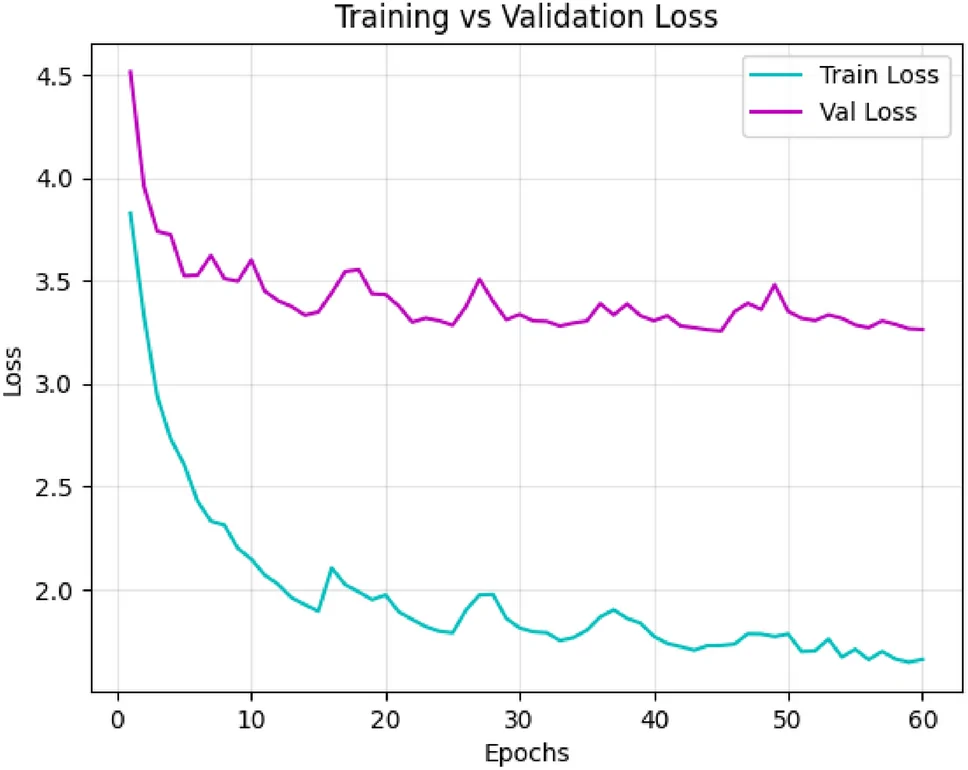
Training and validation loss curves.

The model demonstrates strong performance on several classes, including Accessory tools (F1-score: 94.26%), Normal mucosa and vascular pattern (large bowel) (F1-score: 84.51%), and Normal stomach (F1-score: 91.01%), as shown in Table 5. These categories likely exhibit distinctive visual characteristics, enabling the model to learn discriminative patterns effectively. The integration of EfficientNet-B0 and EfficientNet-B4 backbones facilitates hierarchical feature extraction, allowing the framework to capture both fine-grained local structures and high-level semantic representations. This complementary feature learning contributes to high precision and recall for these well-represented classes.

In contrast, classes such as Colorectal cancer (F1-score: 67.92%) and Resected polyps (F1-score: 30.30%) demonstrate comparatively moderate performance. These categories present higher inter-class similarity and more subtle visual distinctions, making classification more challenging. Although the hierarchical dual-encoder fusion strategy improves feature representation, further enhancement is required for these complex cases. Additionally, Mucosal inflammation (large bowel) (F1-score: 40.00%) shows lower performance, primarily due to limited sample availability and overlapping visual characteristics with related classes.

Overall, despite the presence of class imbalance and varying degrees of visual similarity, the model maintains stable and balanced performance across the 22 categories. A detailed visualization of class-wise predictions is presented in Figure 5, while the training and validation loss curves are illustrated in Figure 6.

### Comparison with existing works

The proposed methodology demonstrates a significant improvement across key metrics such as Micro F1-Score of 0.8411, surpassing both DenseNet-121 and Swin-T, establishes it as the top-performing approach on the GastroVision dataset. The Macro F1-Score of 0.7211 suggests that the methodology effectively balances performance across all classes, including potentially underrepresented ones. An MCC of 0.8191, which is competitive with the best-performing transformer-based approaches, emphasizes its ability to handle the complexities of multi-class classification in GI disease detection.

**Table 7:** Comparison with existing works on the Gastrovision Dataset.

**Models**	**Micro**			**Macro**			**MCC**
	**Precision**	**Recall**	**F1-Score**	**Precision**	**Recall**	**F1-Score**	
Jha, D., Sharma et al. (2023)^41^	0.8203	0.8203	0.8203	0.7388	0.6231	0.6504	0.7987
Subedi, A. et al. (2024)^15^	0.8320	0.8386	0.8324	-	-	-	0.8191
Proposed Methodology	0.8411	0.8411	0.8411	0.7214	0.7330	0.7211	0.8191

### Computational efficiency and inference analysis

Table 8 highlights the strategic trade-offs inherent in the Proposed Hybrid architecture. A key advantage is observed in the computational efficiency; while the standard EfficientNet-B4 requires 9.22 GFLOPs to process its native *380 × 380* resolution, our proposed model reduces this computational burden to 8.02 GFLOPs (a 13% reduction) by optimizing the input dimensions to *320 × 320*. Although the dual-backbone fusion and added CBAM layers slightly increase the parameter count to 20.67M, the reduction in FLOPs ensures the model remains computationally feasible for resource-constrained environments.This efficiency translates directly to competitive inference speeds. While the EfficientNet-B0 baseline offers the lowest latency (1.85 ms) due to its shallow architecture, it lacks the feature extraction capacity required to capture complex gastrointestinal pathologies. In contrast, the Proposed Hybrid model achieves a per-frame latency of 6.50 ms. Although this is marginally higher than the standalone B4 (4.61 ms), it remains well within the critical safety margin for real-time video endoscopy, which typically demands latencies under 33 ms to maintain 30 FPS. Crucially, despite the complex dual-backbone structure, these optimizations allow the Proposed Hybrid to run at 153.81 FPS–approximately *5×* faster than the standard real-time requirement. This confirms the model’s clinical viability, ensuring immediate diagnostic feedback while leaving ample computational headroom for other concurrent clinical software tasks.

**Table 8:** Comparison of computational complexity and inference latency.

**Model**	**Input resolution**	**Params (M)**	**GFLOPs**	**Speed (FPS)**	**Inference time (ms)**
EfficientNet-B0	*224 × 224*	4.34	0.83	540.31	1.85
EfficientNet-B4	*380 × 380*	18.01	9.22	216.75	4.61
Proposed Model	*320 × 320*	20.67	8.02	153.81	6.50

**Table 9:** Ablation study of different backbone combinations.

**Backbone pair**	**Micro**			**Macro**			**MCC**
	**Precision**	**Recall**	**F1-Score**	**Precision**	**Recall**	**F1-Score**	
**Fixed EfficientNet-B0 paired with B0–B4**							
EfficientNet-B0+B0	0.8039	0.8039	0.8039	0.6604	0.7344	0.6874	0.7855
EfficientNet-B0+B1	0.8260	0.8260	0.8260	0.6983	0.7628	0.7240	0.7878
EfficientNet-B0+B2	0.8298	0.8298	0.8298	0.6948	0.7624	0.7223	0.8015
EfficientNet-B0+B3	0.8298	0.8298	0.8298	0.6952	0.7529	0.7166	0.8026
**EfficientNet-B0+B4 (Proposed)**	0.8411	0.8411	0.8411	0.7214	0.7330	0.7211	0.8191
**Fixed EfficientNet-B4 paired with B0–B4**							
**EfficientNet-B0+B4 (Proposed)**	0.8411	0.8411	0.8411	0.7214	0.7330	0.7211	0.8191
EfficientNet-B1+B4	0.8291	0.8291	0.8291	0.6764	0.7486	0.6988	0.7973
EfficientNet-B2+B4	0.8190	0.8190	0.8190	0.6685	0.7459	0.6952	0.7999
EfficientNet-B3+B4	0.8228	0.8228	0.8228	0.6768	0.7820	0.7127	0.7909
EfficientNet-B4+B4	0.8247	0.8247	0.8247	0.6867	0.7761	0.7146	0.7965

**Table 10:** Ablation Study of Fusion Strategies using EfficientNet-B0+B4.

**Fusion strategy**	**Micro**			**Macro**			**MCC**
	**Precision**	**Recall**	**F1-Score**	**Precision**	**Recall**	**F1-Score**	
Late Fusion	0.8373	0.8373	0.8373	0.7052	0.7304	0.7176	0.7981
Concatenation	0.8367	0.8367	0.8367	0.6935	0.7405	0.7162	0.7966
Summation (No CBAM)	0.8329	0.8329	0.8329	0.6933	0.7365	0.7057	0.8047
Summation + Spatial Attention Only	0.8373	0.8373	0.8373	0.7042	0.7369	0.7158	0.8030
Summation + Channel Attention Only	0.8385	0.8385	0.8385	0.7280	0.7202	0.7188	0.8071
**Summation + CBAM (Proposed)**	0.8411	0.8411	0.8411	0.7214	0.7330	0.7211	0.8191

### Ablation study

The results of the extended ablation studies are presented in Tables 9 and 10, evaluating backbone combinations and fusion strategies under a consistent experimental setup. As shown in Table 9, the EfficientNet-B0+B4 configuration achieves the best overall performance, obtaining the highest micro F1-score (0.8411), macro F1-score (0.7211), and MCC (0.8191). Among the fixed EfficientNet-B0 configurations, performance consistently improves as the paired backbone capacity increases from B0 to B4, indicating the benefit of combining lightweight and deeper feature extractors. While EfficientNet-B0+B1 achieves the highest macro F1-score (0.7240) within this subset, its micro performance and MCC remain lower than the proposed B0+B4 configuration. Similarly, EfficientNet-B0+B3 demonstrates competitive macro recall (0.7529), but does not surpass the overall performance of B0+B4.

In the case of fixed EfficientNet-B4 pairings, all alternative combinations (B1+B4, B2+B4, B3+B4, and B4+B4) exhibit lower macro F1-scores and MCC values compared to the proposed configuration. These results confirm that pairing a lightweight backbone (B0) with a higher-capacity backbone (B4) provides complementary representational diversity, effectively balancing fine-grained local feature extraction with high-level semantic representation. This heterogeneous pairing consistently yields superior discriminative performance without introducing unnecessary architectural redundancy.

To further analyze the impact of fusion mechanisms, multiple strategies were evaluated using the finalized B0+B4 backbone configuration as shown in Table10. Late fusion and concatenation yield comparable performance, achieving macro F1-scores of 0.7176 and 0.7162, respectively. Simple summation without attention results in a lower macro F1-score (0.7057) despite a relatively competitive MCC (0.8047). Incorporating individual attention mechanisms improves performance, where Channel attention achieves the highest macro precision (0.7280) and a macro F1-score of 0.7188, while Spatial attention yields a macro F1-score of 0.7158. The proposed summation with CBAM achieves the best overall performance across all metrics, including the highest micro F1-score (0.8411), macro F1-score (0.7211), and MCC (0.8191). This demonstrates that combining both channel and spatial attention within a structured fusion strategy leads to more effective feature refinement and improved class-level discrimination. Overall, the ablation results indicate that both heterogeneous backbone pairing and attention-guided fusion contribute significantly to improved performance, particularly in handling complex and imbalanced multi-class gastrointestinal datasets.

##  Limitations and future work

Despite the strong performance achieved by DualEncoderNet, several limitations should be acknowledged. First, the model was evaluated solely on the GastroVision dataset, and although it includes 22 diverse gastrointestinal categories, variations in imaging protocols, equipment, and patient populations across different medical centers may affect generalizability. Second, the dataset exhibits significant class imbalance, with certain categories containing substantially fewer samples; although class-weighted loss, MixUp augmentation, and macro-averaged evaluation metrics were employed, performance on extremely rare classes remains comparatively lower. Notably, rare-class performance does not correlate strictly with sample count; certain low-support classes with visually distinctive patterns achieve strong F1-scores, whereas visually overlapping categories remain challenging despite moderate sample availability. This indicates that both data imbalance and inter-class similarity influence generalization performance. Third, while the dual-encoder architecture improves hierarchical feature learning, it introduces additional architectural complexity compared to single-backbone models, which may require optimization for deployment in highly resource-constrained environments. Additionally, this study focuses on image-level multi-class classification and does not provide pixel-level lesion segmentation or localization. Finally, real-world prospective validation on live endoscopic video streams and clinical workflow integration was not conducted, which is necessary for full translational deployment.

Future research will focus on validating the proposed framework across independent multi-center gastrointestinal datasets to assess cross-domain robustness and clinical reliability. Advanced imbalance-aware learning strategies, such as focal loss or meta-learning-based reweighting methods, will be explored to further improve minority-class performance. Semi-supervised and self-supervised learning approaches may also be leveraged to utilize large volumes of unlabeled endoscopic data. The hierarchical dual-branch fusion framework can be extended to incorporate decoder modules for simultaneous segmentation and localization, enabling unified classification–segmentation systems. Furthermore, integrating imaging-based phenotypic analysis with genetic, biomarker, or interactome-based computational models represents a promising multimodal direction for understanding multifactorial gastrointestinal diseases. Finally, model compression techniques such as pruning, quantization, or knowledge distillation will be investigated to facilitate deployment in portable and embedded clinical diagnostic systems.

##  Conclusion

In this study, we proposed DualEncoderNet, a new EfficientNet-based hierarchical network for multi-scale gastrointestinal disease detection. By combining two EfficientNet backbones, EfficientNet-B0 and EfficientNet-B4, the framework integrates both local and global features to address the problem of complex GI disease diagnosis. Channel Expansion module and Channel Reduction module work synergistically with CBAM attention to ensure feature integration without information loss. Furthermore, the adoption of SWA and MixUp proved essential in stabilizing the training of the hybrid backbone. The hierarchical feature integration strategy, alongside dual-stage fusion, ensures that the model effectively handles a variety of disease categories, achieving an impressive overall accuracy of 84.11%, Macro-precision 72.14%, Macro-recall 73.30%, and Macro-F1-score 72.11%. The strong performance in micro metrics highlights the model’s ability to consistently identify the most frequent disease categories, while the challenges observed in certain classes, such as colorectal cancer and mucosal inflammation, point to areas for future improvement, particularly related to data limitations and inter-class similarities.

## Data Availability

The dataset used in this study is publicly accessible at https://osf.io/84e7f/(Accessed: 2025-07-25)

## Code availability

The source code for the proposed framework is publicly available at GitHub: https://github.com/testmail9440/gastro. The repository contains the implementation of the dual-encoder architecture, training and evaluation scripts, data preprocessing, and configuration setup necessary to reproduce the experiments reported in this study.
